# SegMine workflows for semantic microarray data analysis in Orange4WS

**DOI:** 10.1186/1471-2105-12-416

**Published:** 2011-10-26

**Authors:** Vid Podpečan, Nada Lavrač, Igor Mozetič, Petra Kralj Novak, Igor Trajkovski, Laura Langohr, Kimmo Kulovesi, Hannu Toivonen, Marko Petek, Helena Motaln, Kristina Gruden

**Affiliations:** 1 Jožef Stefan Institute, Jamova 39, 1000 Ljubljana, Slovenia; 2 University of Nova Gorica, Vipavska 13, 5000 Nova Gorica, Slovenia; 3 Ss. Cyril and Methodius University, 1000 Skopje, Macedonia; 4 University of Helsinki, P.O. Box 68, FI-00014 Helsinki, Finland; 5 National Institute of Biology, Večna pot 111, 1000 Ljubljana, Slovenia

## Abstract

**Background:**

In experimental data analysis, bioinformatics researchers increasingly rely on tools that enable the composition and reuse of scientific workflows. The utility of current bioinformatics workflow environments can be significantly increased by offering advanced data mining services as workflow components. Such services can support, for instance, knowledge discovery from diverse distributed data and knowledge sources (such as GO, KEGG, PubMed, and experimental databases). Specifically, cutting-edge data analysis approaches, such as semantic data mining, link discovery, and visualization, have not yet been made available to researchers investigating complex biological datasets.

**Results:**

We present a new methodology, SegMine, for semantic analysis of microarray data by exploiting general biological knowledge, and a new workflow environment, Orange4WS, with integrated support for web services in which the SegMine methodology is implemented. The SegMine methodology consists of two main steps. First, the semantic subgroup discovery algorithm is used to construct elaborate rules that identify enriched gene sets. Then, a link discovery service is used for the creation and visualization of new biological hypotheses. The utility of SegMine, implemented as a set of workflows in Orange4WS, is demonstrated in two microarray data analysis applications. In the analysis of senescence in human stem cells, the use of SegMine resulted in three novel research hypotheses that could improve understanding of the underlying mechanisms of senescence and identification of candidate marker genes.

**Conclusions:**

Compared to the available data analysis systems, SegMine offers improved hypothesis generation and data interpretation for bioinformatics in an easy-to-use integrated workflow environment.

## Background

Systems biology aims at system-level understanding of biological systems, that is, understanding of system structures, dynamics, control methods, and design methods [1]. Biologists collect large quantities of data from in vitro and in vivo experiments with gene expression microarrays being the most widely used high-throughput platform [2]. Since the amount of available data exceeds human analytical capabilities, technologies that help analyzing and extracting useful information from such large amounts of data need to be developed and used.

The field of *microarray data analysis *has shifted emphasis from methods for identifying individual differentially expressed genes to methods for identifying differentially expressed gene categories (enriched gene sets). A gene set is *enriched *if the member genes are statistically significantly differentially expressed compared to the rest of the genes. One of the most popular controlled vocabularies (ontologies) used for over representation analysis was developed by the Gene Ontology (GO) Consortium [3].

A typical approach to gene set enrichment is to perform Fisher's exact test [4] to identify gene sets annotated by the GO ontology terms which are statistically significantly over-represented. Examples of other approaches include Gene Set Enrichment Analysis (GSEA) [5], GSEA-P [6], Parametric Analysis of Gene set Enrichment (PAGE) [7], and other methods [8-11]. A comparison of several software and web tools (Onto-Express, CLASSIFI, GoMiner, EASEonline, GeneMerge, FuncAssociate, GOTree Machine, etc.) has been performed by Khatri and Draghici [12].

In contrast with the existing gene set enrichment methods, the SEGS (**S**earch for **E**nriched **G**ene **S**ets) semantic subgroup discovery algorithm [13], which is a part of the SegMine methodology, constructs candidate gene sets as combinations of GO terms, Kyoto Encyclopedia of Genes and Genomes Orthology [14] (KEGG) terms, and terms describing gene-gene interactions in the Entrez [15] database. Furthermore, the generalized variant of SEGS called g-SEGS [16] is not limited to the domain of systems biology, and allows for semantic subgroup discovery on any domain using supplied domain ontologies. One way to construct biologically meaningful interpretations from a large amount of experimental data is to present and visualize it using correlation networks. A notable example is ONDEX [17], a database system that combines methods from semantic database integration and text mining with methods for graph-based analysis. It can be applied to the interpretation of gene expression results. Reactome [18], Biocyc [19], BioLayout [20] and MapMan [21] are examples both of curated knowledge bases of metabolic reactions and pathways, and of computational tools to aid in the interpretation of microarrays and similar large-scale datasets. These tools offer powerful techniques for data exploration, but they often are limited to a few types of data and rely on the user to notice relevant connections. In contrast, the Biomine system [22], which is an integral part of SegMine, is a search engine for link discovery and visualization of heterogeneous biological databases. Biomine currently integrates and indexes information from eight major databases (Entrez Gene, UniProt, Gene Ontology, OMIM, NCBI HomoloGene, InterPro, STRING, and KEGG), merged into a single large graph. Moreover, Biomine provides probabilistic graph search algorithms to automatically extract the most relevant subgraphs, and can search for links between given query sets. Due to the complexity of data analysis, bioinformaticians rely more and more on tools that enable composition and reuse of workflows. Several tools exist to support creation, management, and execution of advanced scientific workflows, such as the Taverna workbench [23], the Weka data mining platform [24], KNIME [25], Orange [26], the Kepler scientific workflow system [27], and the Triana problem solving environment [28]. However, workflow environments originating from systems biology have virtually no support for advanced machine learning and data mining techniques, while data mining tools have very limited abilities for making use of the available rich resources of systems biology web services, databases, ontologies and other resources. In contrast, the Orange4WS (Orange for web services) knowledge discovery platform, where the SegMine methodology was implemented, was constructed by integrating data mining software with the wealth of knowledge and services available on the web, including systems biology resources.

The SegMine methodology and its implementation as reusable Orange4WS workflows are the main scientific contributions of this paper. SegMine allows for holistic interpretation of experimental data in the context of general biological knowledge available in public databases. The experimental results from two microarray datasets (a classical acute lymphoblastic leukemia dataset [29] and a dataset on senescence in mesenchymal stem cells [30]) show that SegMine subsumes the results of a state-of-the-art gene set enrichment tool, and can be instrumental in supporting formulation of new hypotheses.

To summarize, this paper presents a new microarray data analysis methodology and its implementation in a newly developed service-oriented workflow environment. It substantially advances previous work in the areas of microarray data analysis [20,21], link discovery and visualization [17,22], and workflow environments [23-26].

## Results and Discussion

This section describes the key results of the presented work. The developed methodology is presented first. Next, the results of the experimental evaluation of the methodology are presented and discussed. Finally, the implementation of the working environment, and the implementation of the methodology itself are described.

### The SegMine methodology

The SegMine methodology aids biologists in interpreting microarray data, in finding groups of genes with semantic descriptions, and in discovering links between them. This leads to better understanding of the underlying biological phenomena and may lead to the formation of new hypotheses, based on the experimental data and biological knowledge available in public databases.

**Figure 1:**
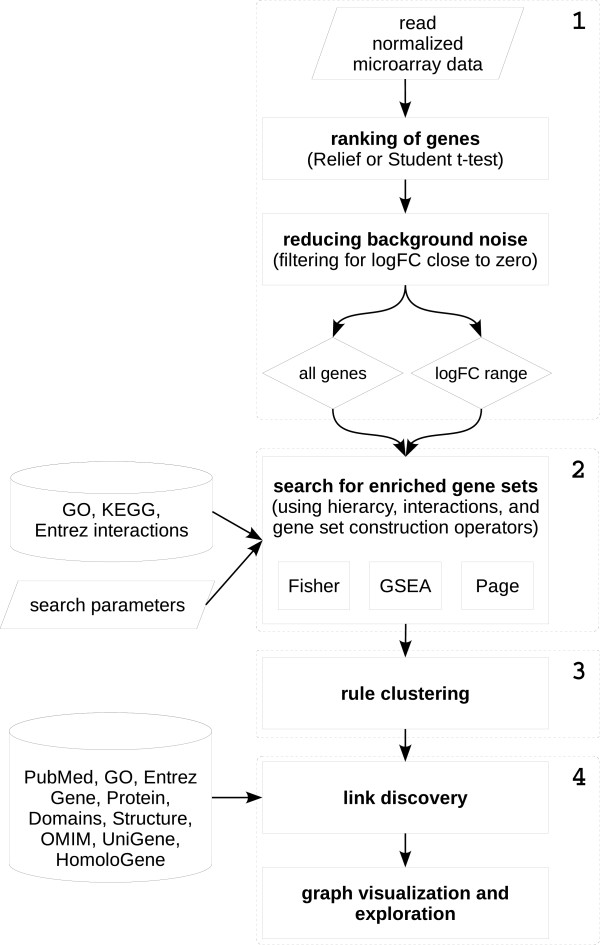
**An overview of the SegMine methodology**. The four main steps of the SegMine methodology: data preprocessing, Identification of differentially expressed gene sets, clustering of rules describing differentially expressed gene sets, and link discovery, graph visualization and exploration. The data preprocesing step (1) takes normalized microarray data as the input, and results in a ranked list of genes. Identification of differentially expressed gene sets (2) is performed by the SEGS algorithm, which makes use of the GO and KEGG ontologies and Entrez interactions to construct gene sets using SEGS operators, hierarchy information, and solution space search parameters. Rules composed of ontology terms describing gene sets that SEGS found to be statistically significant according to three enrichment tests are sent to the agglomerative hierarchical clustering component (3), which enables grouping of similar and separation of different rules. Finally, link discovery and graph visualization (4) is provided by Biomine, which can perform neighbourhood search as well as search for connections between two query sets. Note that SegMine supports the construction of Biomine queries composed of individual genes, gene sets, ontology terms, rules composed of these terms or even whole clusters.

The methodology is based on semantic subgroup discovery with the SEGS algorithm, which is complemented by link discovery and visualization using Biomine services. Several additional steps (e.g. hierarchical clustering, ranking of genes) and features (e.g. resolution of gene synonyms, graph coloring) have been implemented to make the proposed methodology operational and more flexible. A schematic overview of the SegMine methodology is presented in Figure 1.

#### Steps of the SegMine methodology

The SegMine methodology for semantic analysis of microarray data consists of four main steps, which are outlined below. Note that the first two steps can be partially aligned with the general framework for gene set enrichment analysis as proposed by Ackermann et al. [31].

##### 1. Data preprocessing

This step corresponds to the *gene-level statistics *and *transformation *modules of the enrichment analysis framework [31] and is composed of three stages.

In the first stage, SegMine takes class-labeled microarray data that are first loaded and validated as input, and expression fold change (*logFC*) is computed. At this point, different options are available for treating repeated measurements and missing data.

Second, the genes are ranked using the ReliefF [32] algorithm or Student's t-test. Note that other gene-level statistics and methods that result in ranking may also be used, such as fold change, signal-to-noise ratio, correlation networks or Support Vector Machines [31,33-35].

Third, different filtering options can be applied to select a subset of genes. As genes with little variability across samples are often inherently uninteresting, filtering for genes with low |*logFC*| is generally recommended to reduce background noise. Note that the suitable |*logFC*| cutoff point needs to be determined for each dataset separately. Finally, separation of up- and down-regulated genes is also supported.

##### 2. Identifying differentially expressed gene sets

The second step in the SegMine methodology includes the *gene set statistics *and *significance assessment *steps from [31].

The ranked list of genes generated by step one is used as input to the SEGS algorithm [13], which discovers relevant gene groups, described by logical rules formulated as conjunctions of ontology terms from GO, KEGG and Entrez. The rules semantically explain differentially expressed gene groups in terms of gene functions, components, processes, and pathways as annotated in biological ontologies.

SEGS has four main components: (1) the background knowledge (the GO ontology, KEGG pathways annotations, and Entrez interactions), (2) the SEGS hypothesis language (the GO, KEGG and interaction terms, and their conjunctions), (3) the hypothesis generation and pruning procedure utilizing hierarchy relations and solution space search parameters, and (4) statistical evaluation of the hypotheses. Note that SEGS only makes use of the *is_a *and *part*_*of *hierarchical relations in GO.

The SEGS algorithm introduces two new operators, *interact() *and *intersect()*, which can lead to discovery of gene sets that cannot be found by any other currently available gene set enrichment analysis software. If *S *is a gene set and *Entrez *is a database of gene-gene interactions, then the new interacting gene set *INT(S) *is defined as:

(1) $$INT\left(S\right)=\left\{g:\exists g\prime \in S:\exists Entrez\left(g,g\prime \right)\right\},$$

where *Entrez*(*g*, *g'*) is a known interaction of genes *g *and *g' *from the Entrez gene interaction database. Additionally, let *F *be a term from the molecular function branch of the GO ontology, *C *a term from the cellular component branch, *P *a term from the biological process branch, and *K *a KEGG orthology term. Let *F'*, *C'*, *P'*, and *K'*, be the sets of genes annotated by these terms. The new gene set *S *can then be constructed as the intersection of the sets of annotated genes:

(2) $$S_{F,C,P,K}=\left\{g:g\in \left\{F\prime \cap C\prime \cap P\prime \cap K\prime \right\}\right\}$$

The constructed gene sets that are found to satisfy the specified solution space search parameters must be tested for potential enrichment. Currently, SEGS incorporates three different tests commonly used in gene set enrichment analysis: Fisher's exact test, the GSEA method, and parametric analysis of gene set enrichment (PAGE).

The p-values of all three methods may be combined into a single value by taking into account user-defined weights, according to the following formula, which allows for controlling preferences for enrichment tests:

(3) $$p=\frac{\sum w_{i}*p_{i}}{\sum w_{i}}$$

Note that the aggregate p-value is not the p-value in the classical sense but is only used to identify gene sets that have small p-values on several tests.

The significance of gene sets is assessed using permutation testing, but other methods for correcting p-values for multiple hypothesis testing, such as Bonferroni correction or false discovery rate (FDR), can be applied.

##### 3. Rule clustering

The aim of the third step is to reduce the complexity of the results produced by SEGS. Often, several groups of rules found by the SEGS algorithm are composed of very similar gene sets rendering the analysis more difficult due to duplicate information.

Therefore, SegMine incorporates interactive agglomerative hierarchical clustering of SEGS rules to simplify the exploration of large sets of rules, and to provide a natural summarization of the results. Hierarchical clustering of rules is performed according to the similarity of gene sets that are found to be significantly enriched. Several different metrics are available for the computation of similarities, for example, Euclidean, Manhattan, Relief and Hamming. Additionally, agglomerative hierarchical clustering (provided by Orange), supports various linkage criteria for computing clusters including Ward's linkage, complete linkage, single linkage, and average linkage.

##### 4. Link discovery and graph visualization

The last step of the SegMine methodology is provided by the Biomine system, which incorporates several public databases into a single large graph. Biomine implements advanced probabilistic graph search algorithms that can discover the parts of the graph most relevant to the given query. An important integral part of Biomine is the interactive graph visualization component, which supports one click links to the original data sources.

In the Biomine graph data model, nodes of the graph correspond to different concepts (such as gene, protein, domain, phenotype, biological process, tissue), and semantically labelled edges connect related concepts (e.g. gene BCHE encodes protein CHLE, which in turn has the molecular function 'beta-amyloid binding'). The main goal of Biomine is to enable the discovery of new, indirect connections between biological concepts. Biomine evaluates, extracts and visualizes connections between given nodes.

All components of the results from steps 1-3 can be used to formulate queries to the Biomine link discovery engine. SegMine supports the construction of queries composed of individual genes, gene sets, terms from the GO ontology, KEGG pathways, rules composed of these terms, or even whole clusters of gene sets, which are then sent to the Biomine query engine. Biomine is able to find a connecting subgraph between these elements using other entities from a number of public biological databases including Entrez Gene, UniProt, Gene Ontology, OMIM, NCBI HomoloGene, InterPro, STRING, and KEGG pathways. Links in such subgraphs help biologists to uncover unexpected indirect relations and biological mechanisms potentially characteristic for the underlying biological system. Moreover, subgraphs produced by Biomine also present known biological facts, relations, and literature citations in an organized and structured way. Finally, Biomine allows addition of experimental results (e.g., gene expression *logFC *values) to subgraphs, which facilitates the interpretation of discovered links in the context of experimental results.

### Experiments

This section presents two applications of the proposed methodology and its implementation with experimental microarray data. Two microarray datasets were used for the validation and evaluation of the SegMine methodology: a well-known dataset from a clinical trial in acute lymphoblastic leukemia (ALL), and a dataset about senescence in human mesenchymal stem cells (MSC).

### Acute lymphoblastic leukemia

The aim of the first experiment was to validate the SegMine methodology, and perform a comparative analysis of the results using the well-known DAVID [36,37] tool (**D**atabase for **A**nnotation, **V**isualization and **I**ntegrated **D**iscovery). Because DAVID does not provide probabilistic search in large graphs that is provided in SegMine through Biomine services, only the results of the _rst step of the SegMine methodology, namely the sets of differentially expressed genes found by the SEGS algorithm, were used in the comparison.

#### Experimental setup

Comparative analysis was performed on a well-known dataset from a clinical trial in acute lymphoblastic leukemia (ALL) [29], which is a typical dataset for medical research, with several samples available for each class (95 arrays for B-type cells and 33 arrays for T-type cells). This dataset serves as an appropriate reference for such evaluations, as it has also been a model dataset for other microarray data analysis tools [8-10,38,39].

In order to enable direct comparison of the results both tools were set to use the same parameters. The GO ontology, KEGG pathways and Entrez gene-gene interaction database (note that the BIND interaction database was used in DAVID, as DAVID is not able to use Entrez). were used as the background knowledge.

In DAVID, the broadest ontology terms were filtered using the GO FAT filter which attempts to filter the broadest terms (term specificity is based on the number of child terms). On the other hand, a manually created list of terms was used in SegMine.

The top 1000 ranked genes from the data were provided as the input while the remainder (8001) were treated as the background. The resulting enriched terms from DAVID and rules of terms from SegMine were filtered according to the corrected p-value of 0.05. Using DAVID, p-values are obtained with Fisher's exact test and Bonferroni correction. The p-values in SegMine are aggregated by combining p-values of Fisher's exact test, PAGE and GSEA methods, which are corrected using permutation testing. All weights for the aggregation of p-values were equal in our experiments.

**Figure 2:**
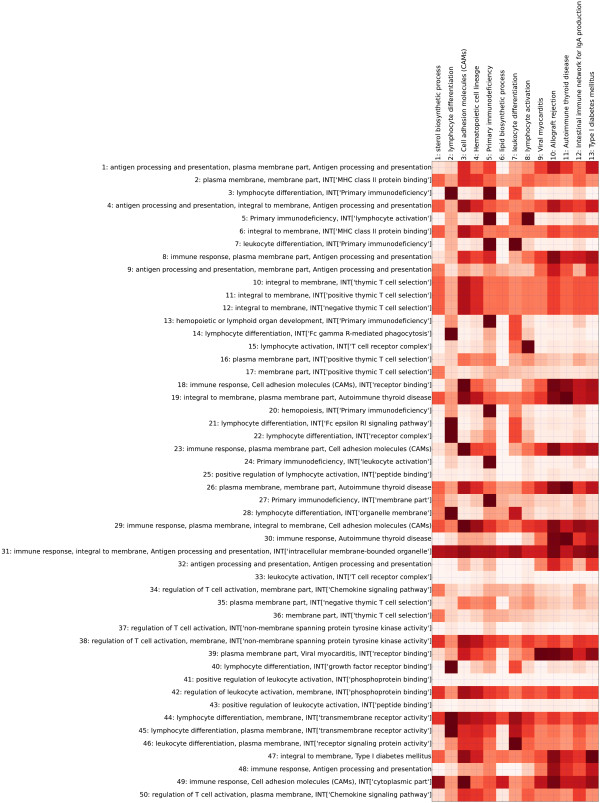
**Comparison of SegMine and DAVID**. The first part of the comparison of the results of SegMine and DAVID on the ALL dataset. Columns are terms found to be enriched by DAVID, while rows are rules of terms found to be enriched by SegMine. Only the first half of the 100 rules of terms obtained by SegMine is shown. All results are statistically significant with *p *≤ 0.05. Darker red shades of matrix cells indicate higher overlapping of corresponding gene sets. Note that rows of the matrix that consist of lightly shaded cells represent gene sets identified as significantly enriched by SegMine but not by DAVID, e.g., 25, 33, 41 and 43.

**Figure 3:**
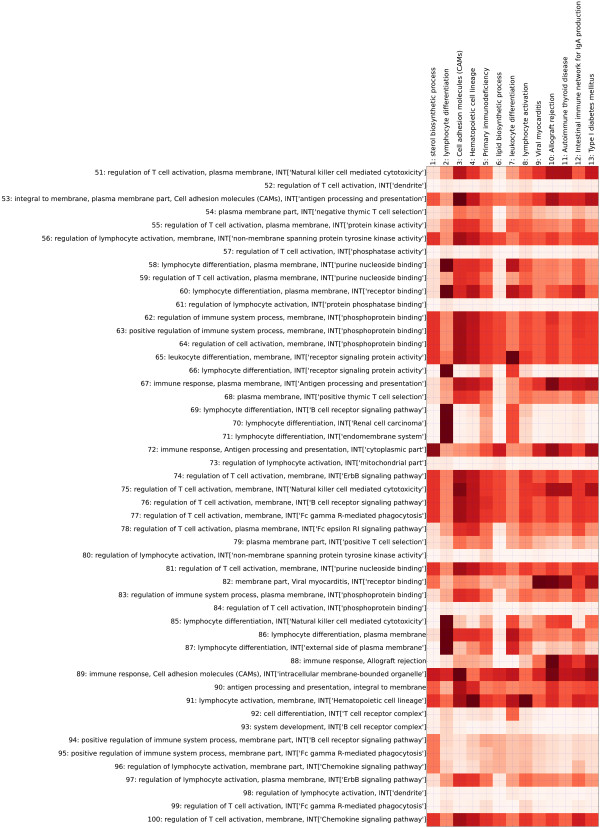
**Comparison of SegMine and DAVID**. The second part of the comparison of the results of SegMine and DAVID on the ALL dataset. Columns are terms found to be enriched by DAVID, while rows are rules of terms found to be enriched by SegMine. The second half of the 100 rules of terms obtained by SegMine is shown. All results are statistically significant with *p *≤ 0.05. Darker red shades of matrix cells indicate higher overlapping of corresponding gene sets. Note that rows of the matrix that consist of lightly shaded cells represent gene sets identified as significantly enriched by SegMine but not by DAVID, e.g., 57, 61, 73, 80, 84, and 98.

As shown in Figures 2 and 3, thirteen terms obtained by DAVID remained after p-value filtering. On the other hand, using SegMine, more rules of terms were found, although only the top 100 are shown. The gene sets covered by DAVID and SegMine were compared using the following formula:

(4) $$v_{i,j}=\frac{\left|S_{i}\cap D_{j}\right|}{\left|D_{j}\right|}$$

where *S_i _*is the set of genes covered by the i-th SegMine rule, and *D_j _*is the set of genes covered by the j-th DAVID term, respectively.

The values *v*_*i*,*j *_∈ [0,...,1] indicate how well the j-th DAVID term is covered by i-th SegMine rule. Note that the exclusion of general terms in SegMine is of key importance for the validity of this measure. If some general terms were found to be enriched by SegMine, according to the above formula they could completely cover gene sets found by DAVID.

Both DAVID and SegMine identified similar enriched gene sets describing differences in gene expression between B-ALL and T-ALL cells, such as lymphocyte differentiation and activation, cell adhesion molecules and KEGG processes in which lymphocyte-specific genes play a major role. Almost all significantly enriched DAVID gene sets were covered by one or more SegMine rules, with the exception of gene set 6 (*lipid biosynthetic process*), which was covered only partially by several SegMine rules (see Figures 2 and 3). The main advantage of the results produced by SegMine is that by combining ontology terms the description of the regulated process is more specific. Many GO terms that were found as enriched by DAVID appear several times in the result of SegMine in conjunction with interacting gene sets.

For example, *lymphocyte differentiation *from the GO ontology appears in 17 SegMine rules in conjunction with different GO and KEGG terms. Such rules can be interpreted as an enrichment of a gene set that includes not only genes described by the first term (*lymphocyte differentiation*) but also interacting genes described by the second term, for example, *Fc gamma R-mediated phagocytosis*.

Additionally, several gene sets obtained by SegMine were not identified by DAVID (Figure 2), for example, rules 25, 33, 41 and 43, which describe *positive regulation of lymphocyte activation *interacting with *peptide binding*, *leukocyte activation *interacting with *T cell receptor complex*, *positive regulation of leukocyte activation *interacting with *phosphoprotein binding *and *positive regulation of leukocyte activation *interacting with *peptide binding*. These rules suggest a different regulation of a set of receptor-interacting genes (or gene products) in the two different lineages of ALL cells.

The comparison shows that SegMine is able to discover the same biological knowledge as DAVID. Moreover, SegMine provides more expressive results in the form of rules, that is, conjunctions of terms. Such rules describe gene sets that are more specific than gene sets reported from other gene set enrichment analysis tools such as DAVID (see Figures 2 and 3), and therefore more suitable for generation of new (more specific) hypotheses. They are evaluated with not only one, but three enrichment tests. Also, the corrected p-values of the available tests can be combined into a single, aggregated value by specifying custom weights controlling user preferences for different gene set enrichment tests.

### Senescence in stem cells

In the second experiment SegMine was applied to the analysis of senescence in human mesenchymal stem cells (MSC). To date, the underlying molecular mechanisms or candidate marker genes that could reflect a degree of cellular aging in MSC are still not known or explained. However, the increasing use of MSC as cellular therapeutics necessitates standardized isolation and reliable quality control assessment of cell preparations. Therefore, we focused on the analysis of a dataset where gene expression profiles from late senescent passages of MSC from three independent donors were compared to the MSC of early passages [30]. We were able to formulate three novel research hypotheses that could improve understanding of mechanisms in senescence and identification of candidate marker genes. One of our hypotheses, derived from the 2008 dataset, may even substantiate a recent proposition independently derived from additional senescence gene expression data [30,40] in 2010. Even though the hypotheses still need to be verified by additional laboratory experiments these results confirm that SegMine is a very useful tool for exploratory analysis of gene expression data and formulation of new research hypotheses.

**Figure 4:**
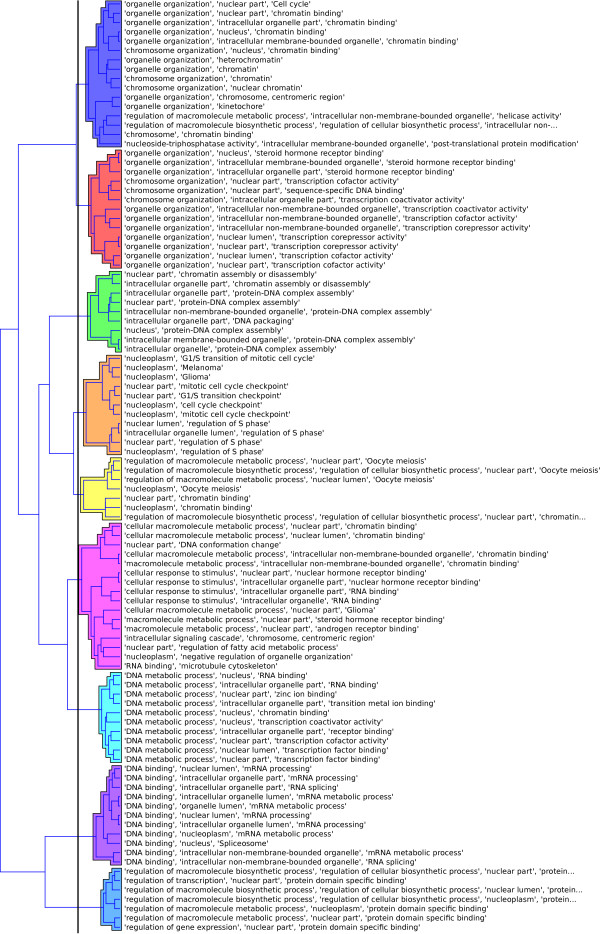
**Hierarchical clusters of rules for the MSC dataset**. Hierarchical clustering of the top 100 statistically significant rules (*p *≤ 0.05). SegMine rules were obtained from a dataset of senescence in human mesenchymal stem cells (MSC dataset). Euclidean distance and Ward's linkage criteria were used to compute the hierarchy.

**Figure 5:**
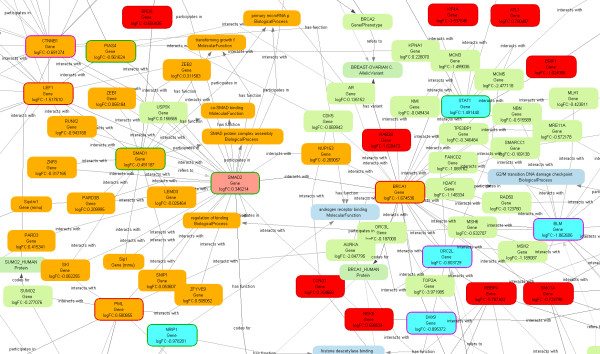
**A SegMine subgraph, where a strongly linked gene hub BRCA1 was identified**. A part of the subgraph obtained by link search between two clusters of rules describing differentially expressed gene sets. All genes covered by the rules from clusters 1 and 9 of hierarchical clustering obtained from the MSC dataset were used. The subgraph allows for the Identification of BRCA1 and SMAD2 gene hubs. Some of the senescent candidate marker genes such as STAT1, MCM3, H2AFX, AURKA, RAD50, MRE11 are linked to the identified BRCA1 gene hub.

**Figure 6:**
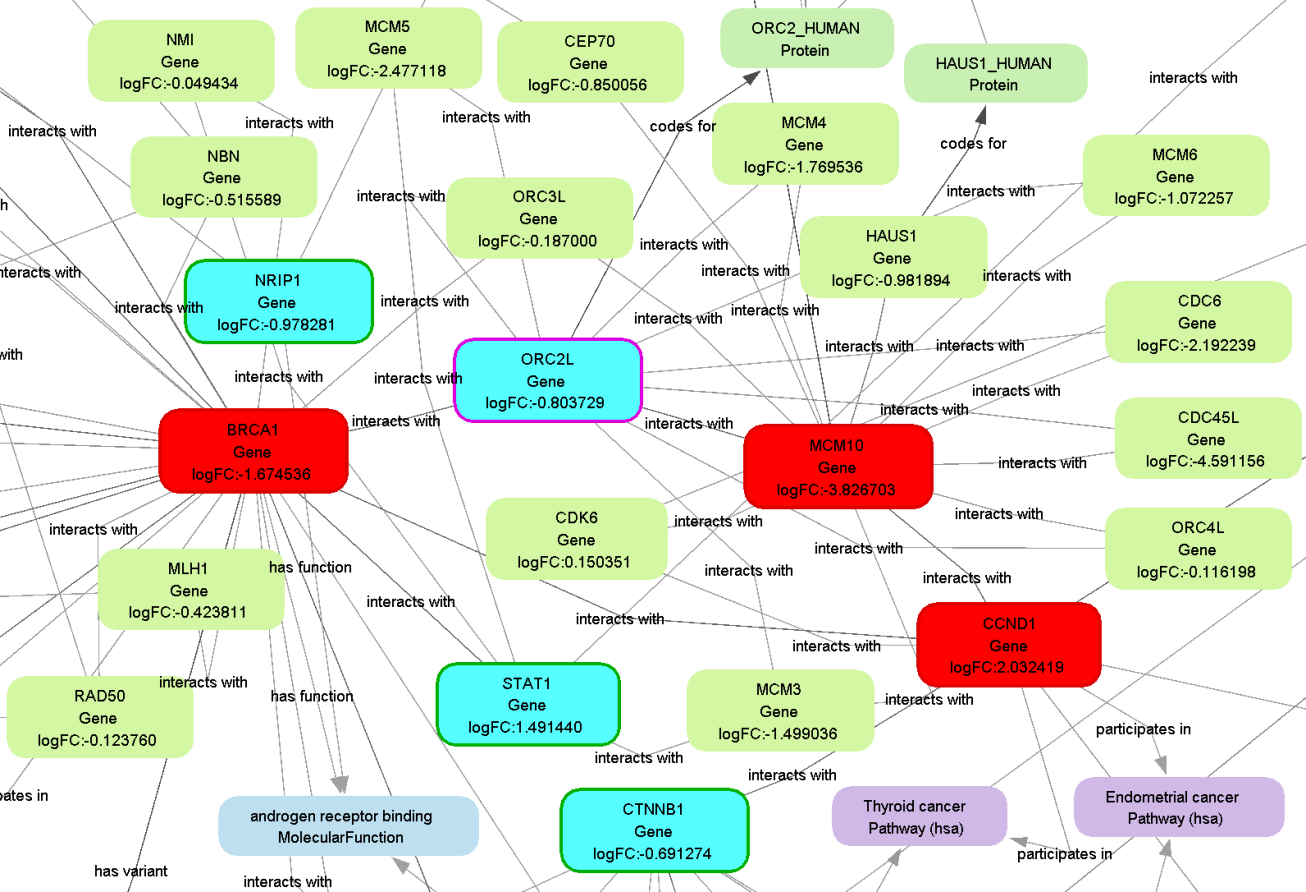
**A SegMine subgraph with the MCM10 gene identified as a gene hub**. A part of the subgraph obtained by link search between two clusters of rules describing differentially expressed gene sets. All genes covered by the rules from clusters 3 and 9 of hierarchical clustering obtained from the MSC dataset were used. The subgraph allows for the Identification of the MCM10 gene hub which is linked to MCM3 and MCM6 senescent candidate marker genes.

Several analyses of microarray data from senescent cells have already been performed [30,40,41]. In these analyses, the senescence candidate marker genes were typically drawn from a list of top differentially expressed genes, that is, their selection depended mainly on their gene expression (*logFC *and p-values). In contrast, SegMine also considers functional properties, as well as direct and indirect connections to related genes and proteins. We have taken the following SegMine steps to analyze the MSC data, published by Wagner et al. [30]:

1. all regulated genes that have absolute *logFC *values lower than 0.3 were filtered out,

2. only SegMine rules with the corrected p-value *p *≤ 0.05 were considered,

3. hierarchical clustering of rules using Ward linkage criteria was used to produce nine rule clusters (Figure 4),

4. several Biomine queries between the source (clusters 1, 2, 3) and target (cluster 9) genes were formulated,

5. the resulting Biomine subgraphs were thoroughly inspected prior to focusing on (a) *gene hubs *(nodes with a large number of edges) where the majority of edges were of the type *interacts with*, and (b) *outlier genes*, which are represented with nodes having few edges with very low weights, or isolated nodes (see Figures 5 and 6).

First we turned attention to the gene enrichment and clustering of rules (steps 2 and 3 above). Comparing these to the originally published results [30], we noticed that our results lack rules annotated with cytoskeletal parts, vacuole or lysosome terms, which had a low number of genes annotated to them in the original study. These compartments are obviously not recognized as important by SegMine. On the other hand, SegMine analysis revealed annotations that were strongly over-represented in Wagner's analysis. We believe that these processes (cell cycle, DNA metabolism and chromatin organization) are indeed crucial for senescence progression. Wagner's group recently approached the same set of senescence associated gene clusters [40] with an improved analysis of additional senescence gene expression data. Similarly to the SegMine clusters, their recent publication does not mention the above unimportant compartments that appeared in [30].

The nine clusters of rules produced in step 3 were further analyzed to find links between distant clusters (step 4 above). In particular, Biomine was queried to discover links between genes from the source clusters 1-3, and the genes of the target cluster 9, respectively. Subgraphs, discovered by Biomine in step 5 were carefully inspected, and the following gene hubs were identified:

1. BRCA1 and SMAD2 genes from the cluster 1 vs. cluster 9 query (Figure 5).

2. SMAD1, SMAD2 genes, and SMARCD1, SMARCE1 genes from the cluster 2 vs. cluster 9 query.

3. MCM10 gene from the cluster 3 vs. cluster 9 query (Figure 6).

Four identified gene hubs (BRCA1 - breast cancer 1, early onset; SMAD2 - SMAD family member 1; SMARCD1 - SWI/SNF related, matrix associated, actin dependent regulator of chromatin, subfamily D, member 1; and MCM10 - minichromosome maintenance complex component 10) were evaluated for the presence of direct links to previously published senescent candidate marker genes. We found some of the senescent candidate marker genes, STAT1 [30], MCM3 [40], H2AFX, AURKA [41], RAD50, and MRE11 [42], to be linked (by the *interacts with *edge) to the BRCA1 gene hub (see Figure 5). Likewise, MCM3 and MCM6 [41] were found to be linked to the MCM10 gene hub (see Figure 6). None of those already identified (patented) senescence candidate marker genes could be recognized as a gene hub by SegMine analysis, as they all had only a limited number of direct links to other genes/proteins. Moreover, a published senescence candidate marker gene SFPQ [41] was even identified as an outlier gene, without any direct link with sufficiently high weight to be present in the Biomine subgraph.

It can be hypothesized that the gene hubs (BRCA1, MCM10, SMAD2, SMARC) identified by SegMine may represent additional senescent candidate marker genes. The results also show that the expression fold difference of genes in gene hubs is not necessarily the highest. We believe that even small expression changes in SMARCD1, SMARCE1 and SMAD2 gene hubs may nonetheless have quite a substantial impact on the process of replicative senescence.

This assumption was confirmed by a literature survey for biological functions of gene hubs identified by SegMine. MCMs, including our newly identified MCM10, have long been known to regulate DNA synthesis by replicative fork formation and to influence proliferation during cells' progression toward senescence, when their expression is switched off [43]. Even BRCA1, a tumor suppressor notorious for its mutation-associated development of certain types of tumors, was recently found to be associated with replicative senescence. The confirmed inactivation of the BRCA1 pathway in MSC was found to reduce their long-term proliferation ability and increase senescence associated beta-galactosidase activity [44]. This functional involvement of the BRCA1/2 and RAD50/MRE11 in replicative senescence, implicated first by Lansdorp [42], was now confirmed also by our SegMine analysis. Additionally, SegMine identified SMADs gene hubs (signal transducers and transcriptional modulators), including SMAD2, which has been known to mediate the TGFb signalling pathway [45] involved in the long-term MSC cultivation resulting in doubling time increase and senescence. Besides senescence, SMADs involved in the TGFb pathways were confirmed to regulate adipogenesis [45]. Similarly, potential involvement of SMARCs genes in adipogenesis was confirmed by profiling of mature differentiated adipocytes vs. proliferatively active adipoblast [46].

As a consequence, we believe that our four identified gene hubs may represent even better senescent gene markers than the patented cell quality markers identified solely by their high expression difference in senescent cells and which were nevertheless found to be connected to our gene hubs (note that only those gene hubs that have edges of type *interacts with *with high probabilities were selected and displayed). Furthermore, SegMine allows visualization of links between genes, enabling clear and easy identification of top processes influencing cellular senescence. Lastly, least, the identification of gene hubs, not necessarily the ones with highest differential expression, allowed us to formulate three new hypotheses (which have yet to be confirmed in future laboratory experiments).

#### Hypothesis 1: Progression to senescence protects cells from entering tumorigenic transition

This hypothesis is Wagner's recent original proposition substantiated with our SegMine results. It was proposed [47] that a central pathway in senescence might provide a purposeful program to protect the organism from tumorigenesis by somatic cells that have accumulated DNA mutations after a certain number of cell divisions. We believe that an additional piece of evidence was revealed to support this hypothesis. Besides the known fact that senescence is not an inevitable fate for all cells, we identified a novel senescence candidate marker gene, the BRCA1 gene hub. The fact that BRCA1 has so far been recognized mostly in tumor development provides additional support for this hypothesis.

#### Hypothesis 2: The Low quality of adipose tissue derived MSC is due to their enhanced tendency to senesce

This hypothesis speculates on a cause for the low quality of adipose derived MSC reported by numerous labs worldwide. Fat derived MSC cease to proliferate and begin to senesce quite early, sometimes even immediately after isolation. SMAD and SMARC gene hubs, identified by SegMine, were all proven in the past to be deregulated during adipogenic differentiation [45,46]. Yet in our analysis they appear also to be over-represented and deregulated in senescent cells; thus we assume that genes of the senescence pathway are most likely involved in adipose tissue homestasis as well. This hypothesis would explain why MSC isolated from the adipose tissue display enhanced permissiveness to senescence upon isolation, as compared to MSC derived from any other tissue.

#### Hypothesis 3: Autophagy may help cells to transiently override their commitment to senesce

Several genes from intracellular protein trafficking and autophagy (MAP1B, LYST, BECN1) were identified by SegMine as outlier genes. When used in Biomine queries they appeared in Biomine subgraphs as nodes with no edges or with edges having very low weights, meaning that knowledge about their links to other genes/proteins is not readily available. However, as cells use autophagy to overcome cell damage or nutrient deprivation, this hypothesis is worth exploring, especially in the light of the SEGS clustering, which on the basis of gene-gene interactions already associated those genes into clusters.

While the above three hypotheses will need to be explored in laboratory experiments to validate their likelihood as contributing factors, the authors believe that SegMine's primary contribution is in providing a unique exploratory environment that allows new hypotheses to be formulated.

### Implementation

In this section we discuss the implementation of the workflow environment named Orange4WS, and the implementation of the methodology itself. Note that the presented implementation of the SegMine methodology is only an example, as it can be implemented very differently in a different environment.

### The Orange4WS data mining platform

The service-oriented data mining platform Orange4WS is an easy-to-use software tool that enables creation and execution of scientific workflows. It is built on top of two open-source projects:

• the Orange data mining framework [26] and

• the Python Web Services project [48].

Orange provides a range of preprocessing, modeling, and data exploration and visualization techniques as well as a user-friendly workflow execution environment. The Python Web Services project enables employment and development of web services using the Python programming language by implementing various protocols and formats including XML [49], SOAP [50] and WSDL [51].

In contrast to other freely available data mining workflow environments such as Weka, Taverna, Triana, KNIME and RapidMiner, the Orange4WS framework offers a unique combination of features: (a) a large collection of data mining and machine learning algorithms, efficiently implemented in C++; (b) a three-layer architecture: C++, Python, and interactive workflows; (c) a collection of very powerful yet easy-to-use data visualization components; (d) incorporation of propositional as well as selected relational data mining algorithms, and (e) simplicity of workflow composition.

As a result, Orange4WS provides a service-oriented data mining software platform, ready to be used for any task requiring data mining algorithms, web services, workflows, complex visualization, rapid prototyping, and other knowledge discovery scenarios. In comparison with the well known Taverna workbench, Orange4WS integrates a complete data mining environment (Orange) with a wide range of machine and data mining algorithms and visualization methods, as well as the ability to use web services and rapid prototyping in Python. Orange4WS offers a high level of abstraction when composing workflows, which contributes to their understandability and simplicity. Finally, Orange4WS also integrates a general knowledge discovery ontology and a planner enabling automated composition of data mining workflows, although this topic is beyond the scope of the work presented here, and therefore will not be discussed. Finally, Orange4WS also enables automated composition of data mining workflows by integrating a general knowledge discovery ontology and a planner, although this topic is beyond the scope of the work presented here, and therefore will not be discussed.

#### Composition and execution of workflows

One of the most important features of Orange, also inherited by Orange4WS, is an easy-to-use interactive workflow construction that is supported by the *Orange Canvas*, an interactive graphical user interface component.

**Figure 7:**
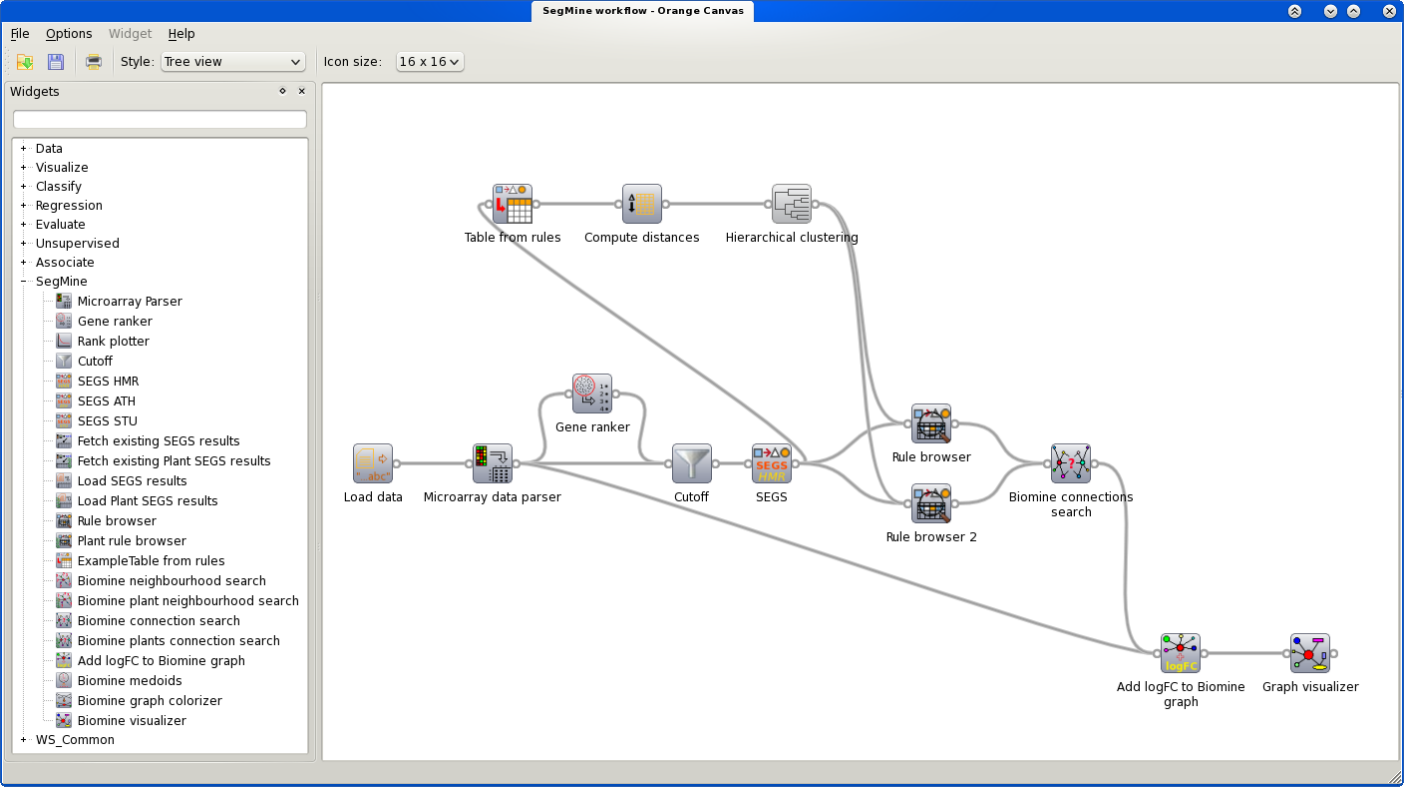
**Orange4WS environment**. A screenshot of Orange4WS running a workflow of SegMine components. The workflow exploits all four main components of SegMine: loading and preprocessing the data, search for enriched gene sets, hierarchical clustering, and link discovery and visualization. All available SegMine, as well as Orange4WS and Orange workflow components, are accessible by clicking on the corresponding items in a tree view on the right.

It enables graphical construction of workflows by allowing interactive workflow elements called *Orange Widgets *to be positioned in a desired order, connected with lines representing flow of data, adjusted by setting their parameters, and finally executed. For example, Figure 7 shows the Orange4WS environment running a workflow of SegMine components (widgets).

The workflow management component enables or disables the connectivity of inputs and outputs according to their types. It also prevents the user from creating loops while connecting widgets by detecting cycles in the corresponding directed graph. If a widget supports the adjustment of its parameters, this can be done from the widget's user interface, which also enables data and results visualization as well as other interactive features. Finally, a constructed workflow can be saved into an XML format that corresponds to a predefined XML schema. This ensures repeatability of scientific experiments as well as support for user collaboration.

Orange4WS offers support for SOAP as well as RESTful web services, which can be used as workflow components. It provides modules that enable:

• loading web service consumer code,

• extracting information about web service input and output data types,

• fully automatic creation of widgets (workflow components) from web services, and

• support for creation of SOAP web services from existing software and algorithm implementations.

When successfully imported, a web service can be used as a normal Orange4WS widget. As a result, Orange4WS enables access to public databases such as PubMed, the BioMart project [52], EMBL-EBI data resources and analysis tools [53], life science web services indexed by BioCatalogue [54], etc.

### SegMine as Orange4WS workflows

We have implemented the SegMine methodology as a collection of Orange4WS workflow components. According to the four steps of the methodology these components can be divided into four groups: (1) data preprocessing, (2) identification of enriched gene sets, (3) rule clustering, and (4) link discovery and visualization.

#### Data preprocessing

The following data preprocessing workflow components (widgets) are available: loading of microarray data from a text file, parsing of microarray data into an internal versatile data structure used by Orange and Orange4WS, resolution of gene synonyms according to the gene data provided by NCBI, ranking of genes using ReliefF algorithm or t-test, loading of precomputed gene ranks from a text file, plotting of gene ranks, and cutoff of ranked genes according to the *logFC *values.

#### Identification of differentially expressed gene sets

Our SegMine implementation offers the following widgets that enable and support identification of differentially expressed gene sets: the SEGS algorithm for different organisms (the current version supports *Homo sapiens*, *Mus musculus *and *Rattus norvegicus *experimental data by integrating corresponding annotations to the ontologies), which is available as a fully SOAP 1.1 compatible web service ready to be used in any service-oriented software supporting SOAP web services, e.g. The Taverna Workflow Management System; a rule browser component, which provides an HTML table visualization where the results are linked to the original data sources; construction of Orange's native data structure from the results of SEGS, which enables the use of data mining techniques and algorithms on the obtained enriched gene sets, and loading and saving the results of SEGS into local files and fetching the results from the server where the SEGS web service is currently running.

#### Rule clustering

Clustering of SEGS rules is provided by the widget for computing distances between rules using different metrics, and the hierarchical clustering widget, which provides different linkage criteria and supports interactive cluster assignment and visualization (see Figure 4). The rule browser component also links the rules to their clusters and provides unions as well as intersections of gene sets in each cluster.

#### Link discovery

The presented implementation offers several components that enable link discovery using Biomine services. First, it provides widgets for neighborhood and connections search as well as search for medoids in a group of genes, all of which query the Biomine web service using the JSON protocol. Second, it integrates the Biomine graph visualization component, which is run locally from Orange4WS as a Java applet. Finally, it implements widgets for adding information about gene expression values, and for coloring selected nodes in Biomine graphs.

## Conclusions

This paper presents SegMine, a methodology for microarray data analysis combining cutting-edge data analysis approaches, such as semantic data mining, link discovery and visualization.

The methodology is implemented in reusable workflows within a new service-oriented data mining platform, Orange4WS. Providing a novel approach to the exploration of microarray datasets in the context of general knowledge is a step beyond the existing state-of-the-art transcriptomic analysis tools. The developed platform is flexible, enabling easy adaptation to the investigated dataset through different filtering options, through different SEGS and Biomine settings, and through different combinations of analysis and visualization tools. The advanced options additionally enable cross-domain link discovery, thus rendering the interpretation of the biological mechanisms underlying differential gene expression understandable to life scientists.

Novel hypotheses, based on the SegMine analysis of MSC microarray data, were presented. We confirmed the strength of SegMine's exploratory analysis, which links the deregulated genes to other related genes/proteins, and this was further supported by literature survey. We were able to formulate three novel research hypotheses that improve understanding of the underlying mechanisms in senescence and identification of candidate marker genes. This may pave the way to a reliable, functionally confirmed panel of senescence marker genes, which can be used as molecular signatures to distinguish between senescent and normal high quality MSC. Such specification of senescence-associated candidate marker genes, functionally evaluated and cross-validated in different MSC preparations, may ultimately result in more reliable quality control of cell preparations, which are increasingly used in cell based therapies.

In the future the presented work will be extended at several levels. While the SegMine methodology is fairly complete, it only provides means for the analysis of genomics data; we plant to extend the methodology to other types of omics data, such as proteomics and metabolomics. The Biomine system currently employs only basic text mining techniques, which will be improved and complemented with natural language processing tools in order to obtain more structured data from textual data sources such as open-access article databases. The SegMine implementation in Orange4WS will be extended with additional components such as visualization of enriched ontology terms similar to the one provided by the GOrilla tool [55]. The Orange4WS workflow environment will also be subject to improvements in order to adapt to the extensions of the methodology, and to improve the support for the publicly available systems biology web services and data and knowledge sources.

## Availability

The Orange4WS platform is available at http://orange4ws.ijs.si.

Our reference implementation of the SegMine methodology for Orange4WS is available at http://segmine.ijs.si.

## Authors' contributions

VP has implemented Orange4WS and SegMine, and contributed to the manuscript. NL and IM conceived and coordinated the computer science aspects of the study and contributed to the manuscript. PKN originated the idea of connecting SEGS and Biomine. IT parallelized and adapted SEGS to be incorporated in SegMine. LL implemented the gene medoids algorithm. KK implemented the Biomine visualizer. HT conceived and coordinated development of Biomine. MP participated in comparison of SegMine and DAVID on the ALL dataset. HM performed an expert analysis of the MSC dataset and formulated new hypotheses. KG coordinated the biological aspects of the study and contributed to the manuscript. All the authors have read and approved the final manuscript.
